# Oxidation‐Enhanced Piezocatalytic Activity in Carbon Nitride‐Based Catalysts for Hydrogen and Hydrogen Peroxide Production

**DOI:** 10.1002/cssc.202500980

**Published:** 2025-08-25

**Authors:** Ying Pan, Luocheng Liao, Xinwen Zhang, Yunya Liu, Ran Su, Nieves López‐Salas

**Affiliations:** ^1^ Department of Chemistry University of Paderborn 33098 Paderborn Germany; ^2^ Key Laboratory of Low Dimensional Materials and Application Technology of Ministry of Education School of Materials Science and Engineering Xiangtan University Xiangtan 411105 China; ^3^ Hebei Key Laboratory of Photoelectric Control on Surface and Interface College of Science Hebei University of Science and Technology Shijiazhuang 050018 P. R. China

**Keywords:** carbon nitride, hydrogen, hydrogen peroxide, piezocatalysis, TiO_2_

## Abstract

Driven by the urgent need for a green, safe, and cost‐effective approach to producing H_2_ and H_2_O_2_—both highly valuable in green energy and environmental protection fields—piezocatalysis, which converts mechanical energy into valuable chemicals, has emerged as a promising solution. However, current catalyst systems face challenges due to the need for materials with both a strong piezoelectric effect and favorable catalytic activity. Herein, the construction of an oxidized carbon nitride (*g*‐C_3_N_4_) matrix anchored with TiO_2_ nanoparticles via alkaline hydrothermal treatment is reported. Under ultrasonication, the *g*‐C_3_N_4_/TiO_2_ composite exhibits optimal performance under carefully controlled alkaline hydrothermal conditions. With a low concentration of Ba(OH)_2_ during hydrothermal treatment, Ba(OH)_2_ provides an alkaline medium, oxidizing the *g*‐C_3_N_4_ species and introducing structural defects into the *g*‐C_3_N_4_ framework. The disruption of the *g*‐C_3_N_4_ matrix, along with its interaction with TiO_2_ nanoparticles, enhances the piezoelectric effect. Consequently, the oxidized *g*‐C_3_N_4_/TiO_2_ composite achieves a remarkable H_2_ production rate of 4427.2 μmol g^−1^ and an H_2_O_2_ production rate of 809.3 μmol g^−1^ within 1 h without the addition of any sacrificial agents or cocatalysts. This work presents an effective strategy for the structural optimization of *g*‐C_3_N_4_‐based materials and may inspire new approaches for designing advanced piezocatalysts.

## Graphical abstract

By harvesting mechanical energy through piezocatalysis, the oxidized g‐C_3_N_4_ loaded with TiO_2_ can directly split pure water into H_2_ or H_2_O_2_. The composite achieves a remarkable H_2_ production rate of 4427.2 μmol g^−1^ h^−1^ and H_2_O_2_ production rate of 809.3 μmol g^−1^ h^−1^.

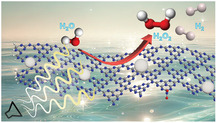

## 1 Introduction

Water serves as a critical resource for biological, industrial, and environmental processes. In addition to its role in sustaining life, water has also emerged as a valuable source of clean energy. The production of hydrogen (H_2_) from water through various catalytic and electrochemical processes is one of the most promising approaches to addressing the growing global energy demand while mitigating environmental concerns associated with fossil fuel consumption.^[^ ^1^ ^]^ As a clean and sustainable fuel, H_2_ holds great potential for applications in fuel cells, energy storage, and industrial chemical processes. Beyond H_2_ production, water can also be utilized to generate hydrogen peroxide (H_2_O_2_), a versatile and environmentally benign oxidant.^[^ ^2^ ^]^ H_2_O_2_ plays a crucial role in the paper and pulp industries, wastewater treatment, medical sterilization, and chemical oxidation processes. Moreover, recent research has highlighted the potential of H_2_O_2_ as an effective liquid fuel in fuel cells, offering a zero‐emission alternative for clean energy generation.^[^ ^3^ ^]^ To develop sustainable methods for H_2_ and H_2_O_2_ production, researchers have explored the conversion of various renewable energy sources, such as solar energy, electrical energy from renewable sources, waste heat, and mechanical vibration, into usable chemical energy.^[^ ^4^ ^]^

Among these approaches, piezocatalysis, a chemical conversion process driven by mechanical vibrations, has been gaining increasing research attention due to its potential for sustainable energy and chemical production. The concept of piezocatalysis was first introduced by Hong et al.^[^ ^5^ ^]^ who demonstrated that piezoelectric fibrous ZnO and dendritic BaTiO_3_ could facilitate the direct decomposition of water into H_2_ and O_2_. This process occurs as mechanical stress induces a piezoelectric field within the material, leading to the separation of electron‐hole pairs. The generated charge carriers then participate in surface redox reactions, enabling catalytic activity without the need for external electrical energy or sunlight. To date, a variety of inorganic catalysts have been developed for H_2_ and H_2_O_2_ production via piezocatalytic processes, including Bi_4_NbO_8_X,^[^ ^6^ ^]^ BaTiO_3_,^[^ ^7^ ^]^ and Zr‐doped HfO_2_.^[^ ^8^ ^]^ While these materials have demonstrated promising catalytic performance, their efficiency in H_2_ and H_2_O_2_ generation remains significantly lower than that achieved through traditional photocatalysis. Further advancements are required to enhance the piezoelectric properties of nano‐ferroelectric materials, enabling more efficient water splitting under ultrasonic vibration.

Graphitic carbon nitride (*g*‐C_3_N_4_), a metal‐free catalyst with a well‐defined layered structure, has garnered significant attention for catalytic applications due to its favorable band edge positions, facile synthesis, and excellent stability.^[^ ^9^ ^]^ The piezoelectric properties of two‐dimensional *g*‐C_3_N_4_ arise from the nanoscale triangular‐shaped non‐centrosymmetric holes, as confirmed through both theoretical calculations and practical experiments.^[^ ^10^, ^11^ ^]^ Additionally, the ring structure of *g*‐C_3_N_4_ provides abundant pyridine nitrogen sites, which have been identified as active centers for oxygen adsorption and activation.^[^ ^12^ ^]^ However, the application of *g*‐C_3_N_4_ as a piezocatalyst for H_2_ and H_2_O_2_ production without the use of sacrificial agents remains largely unexplored. This limitation is primarily due to its weak piezoelectric polarization, which hinders efficient charge separation and catalytic performance. Further research is needed to enhance the piezoelectric properties of *g*‐C_3_N_4_ to fully realize its potential in sustainable energy conversion.

In this study, we present a viable approach for fabricating an alkaline‐treated composite consisting of *g*‐C_3_N_4_ nanosheets and TiO_2_ nanoparticles for the piezocatalytic generation of H_2_ and H_2_O_2_ from water without the use of sacrificial agents or cocatalysts. During the hydrothermal treatment, the alkaline environment facilitates the oxidation of the *g*‐C_3_N_4_ phase and introduces structural defects within the matrix. Benefiting from these structural modifications, the composite achieves a remarkable H_2_ evolution rate of 4427.2 μmol g^−1^ and an H_2_O_2_ production rate of 809.3 μmol g^−1^ under ultrasonication, significantly outperforming the individual components. This work provides valuable insights into the structural engineering of *g*‐C_3_N_4_‐based materials and may inspire further research on the piezocatalytic applications of two‐dimensional materials.

## 2 Results and Discussion

### 2.1 Morphology and Structural Analysis

**Figure 1:**
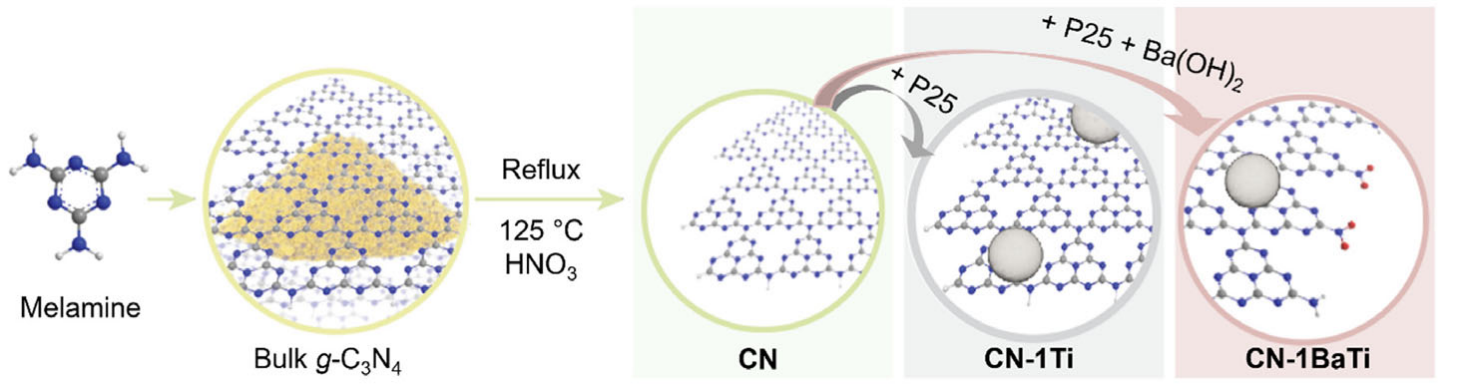
Schematic illustration of the sample preparation process. The colored balls are annotated as: red (oxygen), blue (nitrogen), blue (oxygen), and big gray (loaded nanoparticles).

**Figure 2:**
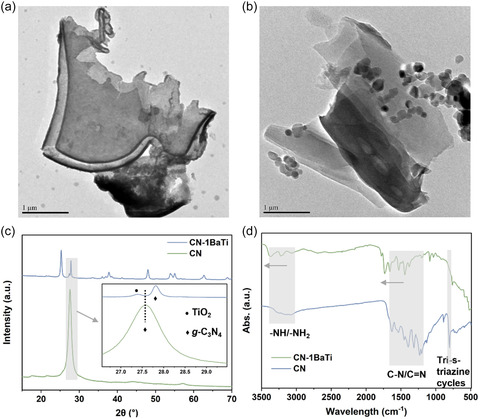
TEM images for a) CN and b) CN‐1BaTi. c) XRD pattern of CN and CN‐1BaTi, inset: enlarged image of the peaks around 27°. d) FTIR patterns of CN and CN‐1BaTi.

**Figure** **1** presents the synthetic strategy for fabricating CN and CN‐BaTi nanosheets. The detailed synthetic parameters can be found in the Supporting Information. In general, CN nanosheets were synthesized via thermal decomposition of melamine, followed by acid exfoliation. The loading of nanoparticles was prepared by mixing CN with varying amounts of TiO_2_ and Ba(OH)_2_, followed by hydrothermal treatment. The morphology and structural characteristics of the samples were analyzed using transmission electron microscopy (TEM) and scanning electron microscopy (SEM). The CN nanosheets exhibit a typical sheet‐like morphology (**Figure** **2**a, S1a, Supporting Information). In contrast, CN‐1BaTi (Figure 2b, S1b, Supporting Information) displays the anchoring of nanoparticles onto the nanosheet surfaces. Furthermore, with increasing Ba(OH)_2_ content, the density of anchored nanoparticles increases, transitioning from a partially decorated state (CN‐2BaTi, Figure S2a,b, Supporting Information) to a nearly complete coverage of the nanosheets (CN‐4BaTi, Figure S2e,f, Supporting Information). TEM coupled with energy‐dispersive X‐ray spectroscopy (EDS) was employed to analyze the elemental distribution within the nanosheet‐nanoparticle structure of CN‐1BaTi (Figure S3 and S4, Supporting Information). The results indicated that the nanosheets were primarily composed of carbon (C) and nitrogen (N), while the nanoparticles were predominantly composed of titanium (Ti) and oxygen (O). Quantitative analysis (Figure S4, Supporting Information) revealed that barium (Ba) was present in negligible amounts.

The phase composition of the samples was characterized using X‐ray diffraction (XRD) and elemental analysis. As shown in Figure 2c, the CN nanosheets exhibit a prominent diffraction peak at 27°, corresponding to the (002) planes of *g*‐C_3_N_4_, which arises from the stacking of conjugated aromatic layers. A weaker peak at 12° is attributed to the (100) plane, associated with the layered packing of tri‐s‐triazine units (JCPDS: 87‐1526).^[^ ^13^ ^]^ The relatively sharp (002) peak suggests that the CN nanosheets possess partial structural order. Upon hydrothermal treatment, the XRD pattern of CN‐1BaTi reveals additional diffraction peaks corresponding to a mixed phase of TiO_2_, including both rutile (JCPDS: 21‐1276) and anatase (JCPDS: 21‐1272), similar to the pattern observed for pure TiO_2_ (P25, Figure S5a, Supporting Information).^[^ ^14^ ^]^ Meanwhile, the intensity of the (100) and (002) peaks of *g*‐C_3_N_4_ decreases, indicating a reduction in the in‐plane ordering of heptazine units, likely due to structural distortion. Additionally, the (002) peak of CN‐1BaTi exhibits a positive shift to a higher diffraction angle, suggesting a decrease in interlayer spacing, which may result from enhanced interlayer interactions. Notably, no Ba‐related diffraction peaks are observed in CN‐1BaTi, indicating that Ba(OH)_2_ and TiO_2_ have not yet formed a detectable amount of BaTiO_3_ under this condition. This is further supported by inductively coupled plasma analysis, which reveals a high Ti content but only trace amounts of Ba in CN‐1BaTi (Table S1, Supporting Information). These results agree well with the TEM‐EDS elemental mapping result. As the BaTi/CN ratio further increases, the (002) peak of *g*‐C_3_N_4_ becomes indistinguishable, likely due to its reduced content and the dominance of sharp diffraction peaks from metal oxides.^[^ ^15^ ^]^ However, the XRD patterns still exhibit TiO_2_ peaks, while new peaks corresponding to BaTiO_3_ emerge (Figure S6a, Supporting Information), indicating that a portion of TiO_2_ has reacted with Ba(OH)_2_ to form BaTiO_3_.

Figure 2 d presents the Fourier transform infrared (FTIR) spectra of CN and CN‐1BaTi. In the spectrum of CN, the broad absorption band observed around 3100–3300 cm^−1^ corresponds to the stretching vibrations of uncondensed primary (—NH) and secondary (—NH_2_) amine groups, which are associated with residual amino functionalities at the edges of CN heterocycles. The absorption peaks in the 1200–1640 cm^−1^ region are attributed to C—N and C=N stretching vibrations within the aromatic units of the CN framework. The characteristic out‐of‐plane bending vibrations of triazine/s‐triazine aromatic units are confirmed by the absorption peaks at 880 and 809 cm^−1^.^[^ ^16^ ^]^ In the case of CN‐1BaTi, the intensity of all these characteristic peaks decreases relative to those of CN, likely due to the relative lower *g*‐C_3_N_4_ content in the composite. With increasing TiO_2_ and Ba(OH)_2_ content in the precursor materials, the intensity of these peaks continues to decrease, as shown in Figure S6b, Supporting Information. Eventually, only peaks corresponding to BaTiO_3_ remain prominent for the pure BTO sample (Figure S5b, S6b, Supporting Information). Furthermore, a shift of these peaks toward higher wavenumbers is observed, which may be attributed to a reduction in bond length within the *g*‐C_3_N_4_ matrix, potentially influenced by changes in the electronegativity of adjacent atoms.

**Figure 3:**
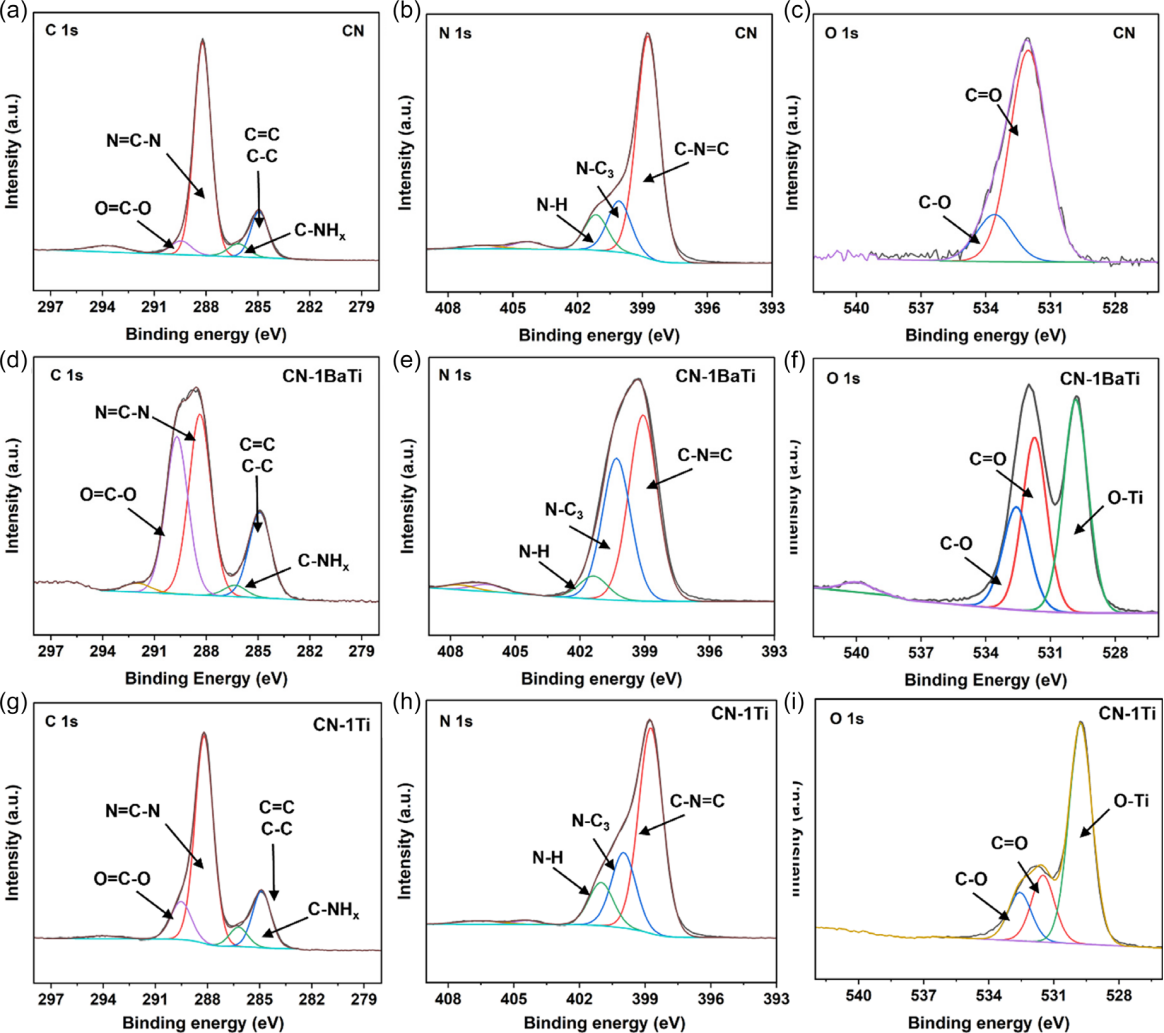
C 1s high‐resolution XPS of a) CN, d) CN‐1BaTi, and g) CN‐1T; N 1s high‐resolution XPS of b) CN, e) CN‐1BaTi, and h) CN‐1Ti; O 1s high‐resolution XPS of c) CN, f) CN‐1BaTi, and i) CN‐1Ti.

X‐ray photoelectron spectroscopy (XPS) analysis was performed to further investigate the interfacial structure of CN and CN‐1BaTi. The survey scan (Figure S7, Supporting Information) confirms the presence of N, C, and O elements in both CN and CN‐1BaTi, while Ti is additionally detected in CN‐1BaTi. A comparison of the high‐resolution C 1s spectra for CN (**Figure** **3**a) and CN‐1BaTi (Figure 3d) reveals four distinct peaks at ≈284.9, 286.1, 288.2, and 289.4 eV, which correspond to adventitious carbon (C—C/C=C), graphitic carbon (C—NH_*x*_), sp^2^‐bonded carbon (N=C—N), and (O=C—O/C—N) coordination, respectively.^[^ ^17^ ^]^ The relative peak intensity of the O=C—O component relative to the N=C—N component significantly increases in CN‐1BaTi, suggesting a structural modification in the *g*‐C_3_N_4_ framework. The high‐resolution N 1s spectra of CN (Figure 3b) and CN‐1BaTi (Figure 3e) exhibit similar features without any additional peaks, indicating that the alkaline hydrothermal treatment promotes the formation of C—O bonds rather than N—O bonds in CN‐1BaTi. The three prominent peaks at ≈398.7, 400.1, and 401.2 eV correspond to sp^2^‐hybridized nitrogen (C—N=C), sp^3^‐hybridized nitrogen (N—(C)_3_), and amino (N—H) functional groups, respectively.^[^ ^18^ ^]^ Notably, the peak intensity of N—(C)_3_ relative to C—N=C increases significantly in CN‐1BaTi, further supporting the structural modifications induced by hydrothermal treatment. In the O 1s spectra (Figure 3c), the peaks at 532.0 and 533.6 eV are attributed to C=O and C—O bonding, respectively. In the CN‐1BaTi sample (Figure 3f), an additional peak emerges at 531.7 eV, which is likely associated with Ti—O bonding. The Ti 2*p* spectrum (Figure S7b, Supporting Information) confirms the presence of Ti—O bonding, which is characteristic of the TiO_2_ phase.^[^ ^15^ ^]^

To further clarify the effects of alkaline treatment on the *g*‐C_3_N_4_ matrix, the chemical bonding states of two comparable samples were prepared: one only added TiO_2_ for the hydrothermal process (CN‐1Ti, Figure 3g–i, S8, Supporting Information), and the other directly mixing CN with TiO_2_ without hydrothermal treatment (M‐1Ti, Figure S9, Supporting Information) were prepared. Without an alkaline environment during hydrothermal processing (CN‐1Ti), the morphology (Figure S8a, Supporting Information) and the phase composition (Figure S8b, Supporting Information) are similar to those of CN‐1BaTi, which is a mixture of *g*‐C_3_N_4_ nanosheets and TiO_2_ nanoparticles. However, the chemical bonding state of CN‐1Ti is similar to that of CN in terms of C (Figure 3g) and N (Figure 3h) species as shown from the C 1s and N 1s spectra and FTIR spectrum (Figure S8c, Supporting Information), whereas the O species are similar to that of CN‐1BaTi because of the TiO_2_ phase in both samples (CN‐1BaTi, CN‐1Ti). Additionally, the relative peak intensity of the O—Ti peak compared to C—O/C=O peaks is much stronger than that in CN‐1BaTi. This may be due to the fact that under the alkaline hydrothermal process, a part of the TiO_2_ nanoparticles were anchored on the surface of the CN matrix, and the rest of the unsuccessfully anchored TiO_2_ were washed away after treatment in an acid solution. Without alkaline conditions or hydrothermal treatment (M‐1Ti), the chemical state of M‐1Ti (Figure S9, Supporting Information) is more similar to that of CN‐1Ti. In summary, the alkaline environment during the hydrothermal process plays a crucial role in modifying the chemical bonding of C and N species within the *g*‐C_3_N_4_ matrix. The alkaline conditions promote the oxidation of the *g*‐C_3_N_4_ phase, leading to the introduction of additional defects within the CN matrix. Additionally, the —OH groups from Ba(OH)_2_ facilitate the anchoring of TiO_2_ onto the CN surface.

### 2.2 Piezocatalytic Activity Analysis

**Figure 4:**
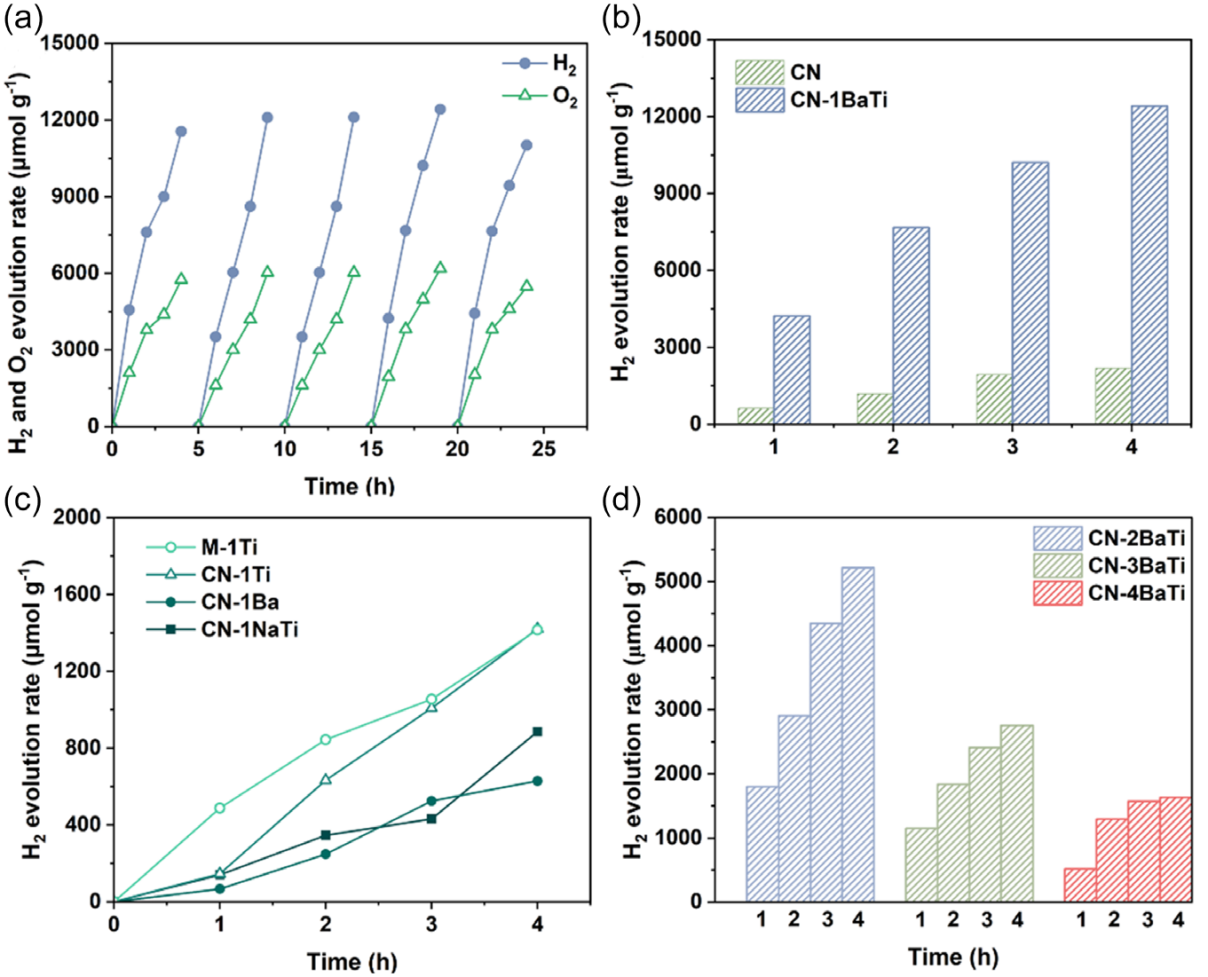
a) Full water splitting recycling tests of CN‐1BaTi under ultrasonication at 40 kHz‐80 W, b) piezocatalytic H_2_ evolution rate of CN and CN‐1BaTi under ultrasonication at 40 kHz‐80 W for 4 h, c) piezocatalytic H_2_ evolution rate of c) CN‐1Ti and M‐1Ti; d) CN‐2BaTi, CN‐3BaTi, and CN‐4BaTi under ultrasonication at 40 kHz‐80 W for 4 h.

To investigate the efficiency of alkaline treatment on the piezocatalytic performance of CN‐1BaTi, the water splitting performance via piezocatalysis was measured in pure water under 40 kHz ultrasonic vibration. As shown in **Figure** **4**d, CN‐1BaTi demonstrates excellent piezocatalytic stability, with no significant degradation in H_2_ and O_2_ production rates over four consecutive cycles. As expected, the mole ratio of H_2_ and O_2_ evolution is nearly 2:1, showing a full water splitting mechanism: 2H_2_O → 2H_2_ + O_2_. CN‐1BaTi exhibits a significantly higher H_2_ evolution rate compared to CN over a 4 h ultrasonic treatment (Figure 4b). Specifically, the H_2_ evolution rate reaches 4427.2 μmol g^−1^ for CN‐1BaTi, whereas CN achieves only 621.9 μmol g^−1^ in 1 h. To isolate the effect of pH value and confirm the unique effect of Ba(OH)_2_ and TiO_2_, four control samples were conducted: the H_2_ production rates of CN‐1Ti (*g*‐C_3_N_4_ + TiO_2_), CN‐1Ba (*g*‐C_3_N_4_ + Ba(OH)_2_), CN‐1NaTi (*g*‐C_3_N_4_ + NaOH + TiO_2_), and M‐1Ti (*g*‐C_3_N_4_ physically mixed with TiO_2_) were measured under the same conditions. As shown in Figure 4c, the H_2_ evolution rates of these control samples for 1 h were 146.0 μmol g^−1^ (CN‐1Ti), 66.7 μmol g^−1^ (CN‐1Ba), 140.1 μmol g^−1^ (CN‐1NaTi), and 487.2 μmol g^−1^ (M‐1Ti), respectively. These values are all significantly lower than that of CN‐1BaTi, highlighting the distinct advantage conferred by the oxidative environment provided by Ba(OH)_2_ prior to its reaction with TiO_2_ to form the BaTiO_3_ phase. More detailed structure‐performance correlation of the control samples was explained in Figure S10, Supporting Information.

To further assess the contribution of Ba‐based species, the H_2_ evolution rates of CN‐2BaTi, CN‐3BaTi, CN‐4BaTi, and pristine BTO were also measured for comparison (Figure 4d, S11, Supporting Information). Within 1 h, the H_2_ production rates of CN‐BaTi, CN‐3BaTi, CN‐4BaTi, and pristine BTO were 1800.7, 1150.5, 517.8, and 77.5 μmol g^−1^, respectively, all of which were lower than that of CN‐1BaTi. The H_2_ evolution rate of the mixtures decreased with an increasing proportion of Ba‐based species (BaTiO_3_), indicating a possible inhibitory effect on the piezocatalytic activity.

Our structural analysis and catalytic comparisons confirmed that the physical mixing of *g*‐C_3_N_4_ and TiO_2_ did not enhance the catalytic performance of the individual components. This is because pristine *g*‐C_3_N_4_ and TiO_2_ exhibited low piezocatalytic performance due to their insufficient piezoelectric potential.^[^ ^19^, ^20^ ^]^ Following hydrothermal treatment, the small amount of Ba(OH)_2_ introduced during the hydrothermal process created an alkaline environment, which oxidized the *g*‐C_3_N_4_ nanosheets, generating more defects within the CN matrix. This oxidation not only disrupted the regular arrangement of the *g*‐C_3_N_4_ matrix, thereby improving the catalytic activity,^[^ ^16^ ^]^ but also facilitated the anchoring of TiO_2_ species onto the CN matrix.^[^ ^21^ ^]^ As the amount of Ba(OH)_2_ increased during hydrothermal treatment, a portion of TiO_2_ reacted with Ba(OH)_2_, leading to the formation of a composite system consisting of *g*‐C_3_N_4_, TiO_2_, and BaTiO_3_ phases. Previous studies have demonstrated that bulk BaTiO_3_ exhibits low piezocatalytic performance due to its insufficient piezoelectric potential.^[^ ^22^ ^]^ Consequently, an increased presence of BaTiO_3_ resulted in the agglomeration of the nanoparticles and the shielding of active sites on the CN matrix, leading to a decline in piezocatalytic performance. The optimal catalytic activity was achieved when an optimal amount of Ba(OH)_2_ was introduced, corresponding to the CN‐1BaTi composition.

**Figure 5:**
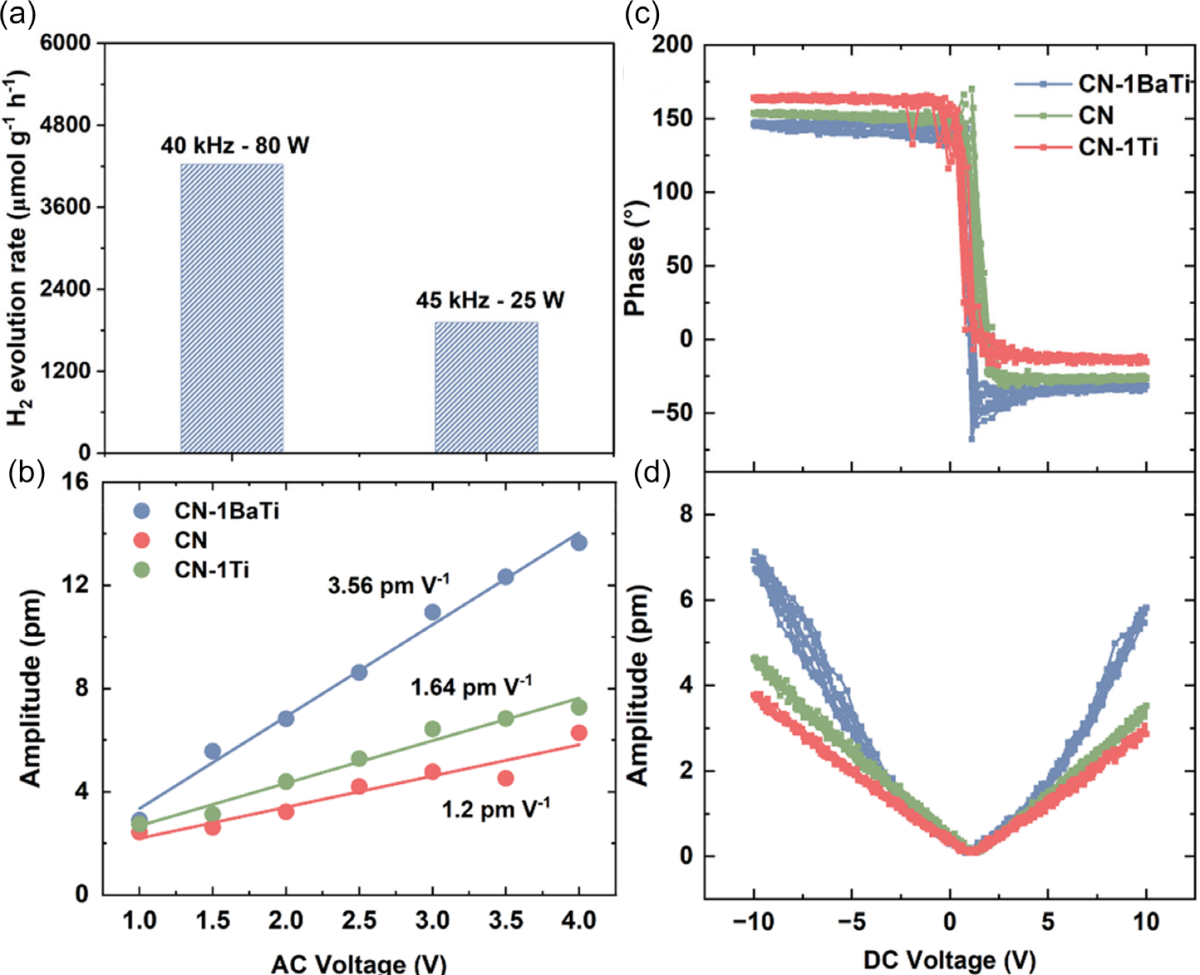
a) Piezocatalytic H_2_ evolution rate of CN‐1BaTi under different ultrasonication powers for 1 h; b) The average piezoresponse amplitude as a function of excitation AC voltage for CN‐1BaTi, CN, and CN‐1Ti via SS‐PFM; c) phase and d) amplitude hysteresis loops for CN‐1BaTi, CN, and CN‐1Ti.

The effect of ultrasonic power on the piezocatalytic H_2_ production performance was investigated at a frequency of 40 kHz under ultrasonic powers of 25 and 80 W. As shown in **Figure** **5**a, the H_2_ production rate decreased with decreasing ultrasonic power. This trend can be attributed to the fact that higher ultrasonic power induces greater deformation and generates a stronger internal electric field, which enhances charge carrier separation and improves catalytic performance. However, a more comprehensive study examining both frequency and power is necessary to determine the optimal ultrasonication conditions. The average piezoresponse amplitude as a function of excitation AC voltage is demonstrated in Figure 5b, which shows a typical linear relationship, substantiating the piezoelectric behavior of these three samples. The linear fitting further provides the effective piezoelectric coefficient to be 1.9, 1.64, and 3.56 pm V^−1^ for CN‐1BaTi, CN, and CN‐1Ti, respectively. CN‐1BaTi has the highest piezoresponse among all three samples. It should be noted that the piezoresponse has been calibrated, with the amplification factor at the resonance frequency deducted. Details are provided in Figure S13, Supporting Information. Meanwhile, typical ferroelectric switching behavior has been revealed by the switching spectroscopy piezoresponse force microscopy (SS‐PFM) that shows a complete reversal of 180° in the phase signals (Figure 5c) and a corresponding typical butterfly (Figure 5d) at the minimum of the amplitude signals.

**Figure 6:**
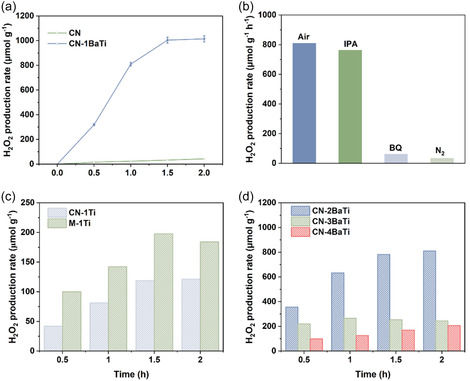
a) Piezocatalytic H_2_O_2_ production rate of CN and CN‐1BaTi under ultrasonication at 40 kHz‐80 W for 2 h, b) piezocatalytic H_2_O_2_ production rate of CN‐1BaTi under different conditions; piezocatalytic H_2_O_2_ production rate of c) CN‐1Ti and M‐1Ti; d) CN‐2BaTi, CN‐3BaTi, and CN‐4BaTi under ultrasonication at 40 kHz‐80 W for 2 h.

The piezocatalytic H_2_O_2_ production performance of CN and CN‐1BaTi was also evaluated in pure water. As shown in **Figure** **6**a, the amount of H_2_O_2_ increased over time for both samples. Notably, CN‐1BaTi exhibited a significantly higher H_2_O_2_ production rate compared to CN. Within 1 h, the H_2_O_2_ production rate of CN‐1BaTi reached 809.3 μmol g^−1^, ≈34 times higher than that of CN (24.0 μmol g^−1^). This result is also significantly higher than the values reported in the literature (Table S2, Supporting Information). To investigate the mechanism underlying the enhanced piezocatalytic performance of CN‐1BaTi, a series of controlled experiments was conducted.^[^ ^23^ ^]^ The role of oxygen in the piezocatalytic reaction was examined by varying the atmospheric conditions during H_2_O_2_ production (Figure 6b). Under an air atmosphere, the H_2_O_2_ yield reached 809.3 μmol g^−1^ within 1 h. However, when the reaction was performed under a nitrogen atmosphere, H_2_O_2_ generation significantly decreased, indicating that O_2_ from the air is the primary source of H_2_O_2_ via reduction. This suggests that the contribution of water oxidation to H_2_O_2_ production is minimal during the piezocatalytic process.^[^ ^24^ ^]^ To further identify the active species involved, superoxide radical (•O_2_^−^) and hydroxyl radical (•OH) scavengers were introduced. Benzoquinone (BQ), a •O_2_^−^ scavenger, and isopropanol (IPA), a •OH scavenger, were added to the reaction system. As shown in Figure 5b, the addition of BQ significantly reduced H_2_O_2_ production, indicating that •O_2_^−^ plays a crucial role in the piezocatalytic process. In contrast, the addition of IPA had a negligible effect on H_2_O_2_ generation, suggesting that •OH is not involved in the reaction. Based on these findings and previous reports, it can be concluded that CN‐1BaTi facilitates H_2_O_2_ production via a two‐electron oxygen reduction pathway (O_2_ → •O_2_^−^ → H_2_O_2_).^[^ ^25^ ^]^

The H_2_O_2_ production rates of various control samples were also measured. Consistent with the H_2_ evolution results, the absence of an alkaline environment (CN‐1Ti) or hydrothermal treatment (M‐1Ti) led to significantly lower H_2_O_2_ yields. Within 1 h, CN‐1Ti and M‐1Ti (Figure 6c) produced only 81.2 and 100.0 μmol g^−1^ of H_2_O_2_, respectively, compared to the significantly higher yield of CN‐1BaTi (809.3 μmol g^−1^). Furthermore, as the amount of Ba(OH)_2_ introduced during hydrothermal treatment increased, the H_2_O_2_ production rate gradually declined due to the increasing presence of BaTiO_3_ (Figure 6d, S13, Supporting Information). The H_2_O_2_ yields for CN‐2BaTi, CN‐3BaTi, CN‐4BaTi, and pristine BTO were 632.6, 265.4, 125.9, and 57.0 μmol g^−1^, respectively. These results confirm that CN‐1BaTi is the optimal sample for both H_2_ and H_2_O_2_ production from pure water.

## 3 Conclusion

In summary, we report a piezoelectric catalytic composite consisting of alkaline‐treated *g*‐C_3_N_4_ nanosheets and TiO_2_ nanoparticles. The optimized sample, CN‐1BaTi, exhibited the highest performance in both hydrogen and hydrogen peroxide production from pure water without adding any sacrificial agents or cocatalysts, achieving yields of 4427.2 and 809.3 μmol g^−1^, respectively. The introduction of a small amount of Ba(OH)_2_ during the hydrothermal process created an alkaline environment that oxidized the *g*‐C_3_N_4_ nanosheets, generating defects within the CN matrix. This oxidation not only disrupted the regular arrangement of the *g*‐C_3_N_4_ structure, thereby enhancing the piezoelectric effect, but also facilitated the anchoring of TiO_2_ species onto the CN matrix. We anticipate that our findings on catalyst preparation and the systematic optimization of piezoelectric systems will provide valuable insights into the design of next‐generation high‐performance piezoelectric materials.

## Conflict of Interest

The authors declare no conflict of interest.

## Author Contributions

**Ying Pan**: conceptualization, data curation, formal analysis, investigation, visualization, methodology, and writing. **Luocheng Liao** and **Yunya Liu**: piezoelectric effect testing and data analysis. **Xinwen Zhang**: H_2_ quantification. **Ran Su**: data curation, resources, and formal analysis. **Nieves López‐Salas**: formal analysis, visualization, and resources. All authors contributed to discussions and commented on the manuscript, and all authors reviewed the manuscript.

## Supporting information

Supplementary Material

## Data Availability

The data that support the findings of this study are available from the corresponding author upon reasonable request.
