# Supplementary material for: Oxidation‐Enhanced Piezocatalytic Activity in Carbon Nitride‐Based Catalysts for Hydrogen and Hydrogen Peroxide Production
Source: ChemSusChem. 2025 Aug 25;18(19):e202500980. doi: 10.1002/cssc.202500980 (PMC12487744; doi:10.1002/cssc.202500980)
Supplement: Supplementary file 1 — Supplementary Material [file CSSC-18-e202500980-s001.pdf]

Supporting information

Oxidation-Enhanced Piezocatalytic Activity in Carbon Nitride-Based Catalysts for Hydrogen and Hydrogen Peroxide Production

Ying Pan,[a,*] Luocheng Liao,[b] Xinwen Zhang,[c] Yunya Liu,[c] Ran Su,[b,*] Nieves Lopez Salas[a,*]

[a] Department of Chemistry, University of Paderborn, Paderborn 33098, Germany

[b] Key Laboratory of Low Dimensional Materials and Application Technology of Ministry of Education, School of Materials Science and Engineering, Xiangtan University, Xiangtan 411105, China

[c] Hebei Key Laboratory of Photoelectric Control on Surface and Interface, College of Science, Hebei University of Science and Technology, Shijiazhuang 050018, P. R. China

ying.pan@uni-paderborn.de; suranxida@163.com; nieves.lopez.salas@uni-paderborn.de

Experimental Section

Materials

Hydrogen peroxide (H_2O_2, 35%), hydrochloric acid (HCl, 37%), melamine ($C_3H_6N_6$, > 98%, TCI), potassium iodide (KI, > 99.5%, Merck Darmstadt), ammonium heptamolybdate tetrahydrate ($H_{32}Mo_7N_6O_{28}$, > 99%), 1,4-Benzoquinone ($C_6H_4O_2$, > 98%, Acros Organics), 2-propanol (C_3H_8O), potassium chloride (KCl, > 99%, Fisher), titanium oxide (TiO_2 P25, Merck), barium hydroxide ($Ba(OH)_2$, TCL, > 98%). All chemicals are used in experiments without further purification.

Samples preparation

Preparation of CN: Melamine powder was placed in a covered crucible and heated in a furnace at 600 °C for 2 h under N_2 atmosphere at a heating rate of 3 °C min^{-1}. The furnace was then cooled down naturally, and the yellow product obtained was collected and milled into fine powder. After that, 1 g of the fine powder was refluxed at 125°C for 24 h in an HNO_3 aqueous solution (5M, 150 mL). Then the powder was washed with water 5 times to remove HNO_3 and dried at 90 °C for 12 h. The final product was named CN.

Preparation of CN-BaTi: 0.3 g of CN, a certain amount (1, 2, 3, 4 mmol) of P25, and a certain amount (2, 4, 6, 8 mmol) of $Ba(OH)_2$ were mixed in 60 mL of water. The mixture was transferred into a 100 mL Teflon-lined autoclave and maintained at 180 °C for 24 h. After naturally cooling down to room temperature, the formed products were centrifuged and washed with 1 M HCl and water 3 times and then dried at 90 °C overnight. The obtained products were named according to the amount of P25 and $Ba(OH)_2$. "Ba" stands for $Ba(OH)_2$, and "Ti" stands for P25. "1BaTi" indicates that 1 mmol of P25 and 2 mmol of $Ba(OH)_2$ were added during the catalyst preparation stage. "2BaTi", "3BaTi", and "4BaTi" correspond to twofold, threefold, and fourfold increases, respectively, in the amounts of P25 and $Ba(OH)_2$ compared to "1BaTi." So, CN-1BaTi means (0.3g CN + 1 mmol P25 + 2 mmol $Ba(OH)_2$), CN-2BaTi (0.3g CN + 2 mmol P25 + 4 mmol $Ba(OH)_2$), CN-3BaTi (0.3g CN + 3 mmol P25 + 6 mmol $Ba(OH)_2$), and CN-4BaTi (0.3g CN + 4 mmol P25 + 8 mmol $Ba(OH)_2$).

Preparation of BTO: A mixture of 10 mmol of P25 and 20 mmol of $Ba(OH)_2$ was dispersed in 60 mL of water. The subsequent steps followed the same procedure as the preparation of CN-BaTi.

Preparation of CN-1Ti: A mixture of 0.3 g of CN and 1 mmol of P25 was dispersed in 60 mL of water. The subsequent steps followed the same procedure as the preparation of CN-BaTi.

Preparation of CN-1Ba: A mixture of 0.3 g of CN and 2 mmol of $Ba(OH)_2$ was dispersed in 60 mL of water. The subsequent steps followed the same procedure as the preparation of CN-BaTi.

Preparation of CN-1NaTi: A mixture of 0.3 g of CN, 1 mmol of P25, and 4 mmol of NaOH was dispersed in 60 mL of water. The subsequent steps followed the same procedure as the preparation of CN-BaTi.

Preparation of M-1Ti: A mixture of 0.3 g of CN and 1 mmol of P25 was dispersed in 60 mL of water. The mixture was then stirred at room temperature for 24 h. After that, the mixture was centrifuged and washed with 1 M HCl and water 3 times and then dried at 90 ℃ overnight.

Materials Characterization

The crystallization of the samples was examined by an X-ray Diffractometer (XRD, Shimadzu 700) with a Cu-Kα radiation (λ = 0.154 nm) in the range of 2° - 80°. The bonding states of the samples were tested by using a Fourier-transform infrared spectroscopy (FTIR, Spectrum Two, Perkin Elmer GmbH) in the range of 4000-450 cm^{-1}. The microstructure of the samples was observed by the Scanning electron microscope (SEM, Zeiss Neon 40) instrument to characterize the surface morphologies and microstructures of the samples. The high-resolution images of the samples were taken by transmission electron microscopy (TEM, JEOL ARM200CF). The elemental mapping of the samples was conducted by using a field-emission transmission electron microscope equipped with an energy dispersive spectroscopy (TEM-EDS, FEI Talos F200S). The composition of the non-metal elements was tested by a MICRO cube CHNOS elemental analyzer (Elementar Analysensysteme GmbH). The compositions of the metal elements were tested by Inductively coupled plasma mass spectrometry (ICP-MS, ARL Fisons SS-7).

Piezoelectric effect testing

All measurements were performed by a commercial Asylum Research MFP-3D system at room temperature under ambient conditions. For SSPFM measurements, commercial silicon tips with conductive Ti/Ir coating (ASYELEC.01-R2, Asylum Research) were used. In the PFM measurements, a conductive ITO substrate was used and properly grounded to minimize potential measurement artifacts. The inverse optical lever sensitivity has been calibrated, and the Q factor of each sweeping curve at the resonance frequency has been subtracted to obtain the effective piezoresponse coefficients.

Piezocatalytic performance testing

Piezocatalytic production of hydrogen: In a run of piezo-catalytic water-splitting hydrogen production, 10 mg of catalyst powder was dispersed in 30 mL of pure water, bubbled with Ar, and sealed with parafilm. The suspension was exposed to ultrasonic vibration. The temperature of the piezo-catalytic system was controlled to be 20 °C using a cooling system. The gas produced was periodically withdrawn

with a syringe and examined by gas chromatography (GC).

Piezocatalytic production of hydrogen peroxide: In a run of piezo-catalytic water-splitting hydrogen production, 10 mg of catalyst powder was dispersed in 25 mL of pure water in a reactor without sealing. The suspension was exposed to ultrasonic vibration. The temperature of the piezo-catalytic system was controlled to be 17 °C using a cooling system. The suspension was exposed to ultrasonic vibrations. Samples collected at different reaction times were centrifuged for the absorbance measurements. 500 µL suspension at different reaction times after centrifugation was mixed with 2 mL 0.1 mM KI solution and 50 µL 0.01 mM $H_{32}Mo_7N_6O_{28}$ solution. The concentration of H_2O_2 was determined by analyzing the absorbance of the mixed solution at 352 nm after diluting it five times on an Ultraviolet-visible spectroscopy (UV-Vis, PerkinElmer Lambda).

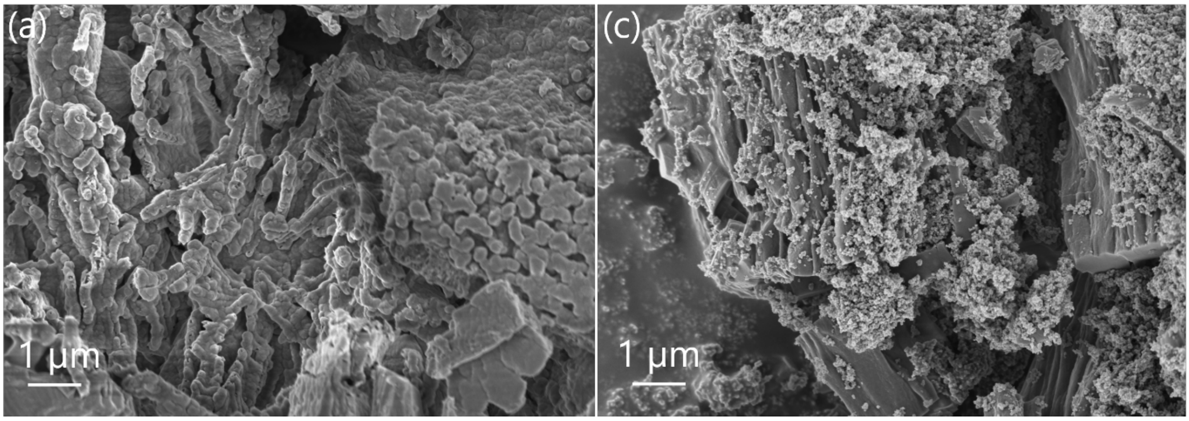

Fig. S1. SEM images for (a) CN and (b) CN-1BaTi.

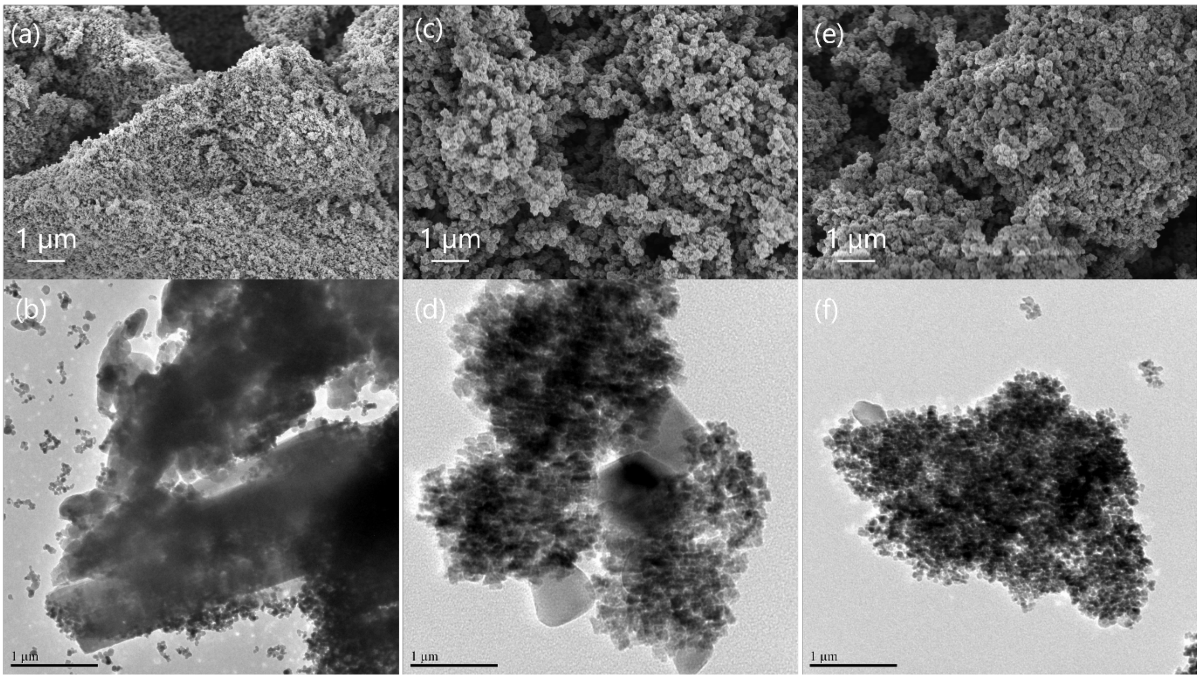

Fig. S2. SEM images for (a) CN-2BaTi, (c) CN-3BaTi, and (e) CN-4BaTi; TEM images for (b) CN-2BaTi, (d) CN-3BaTi, and (f) CN-4BaTi.

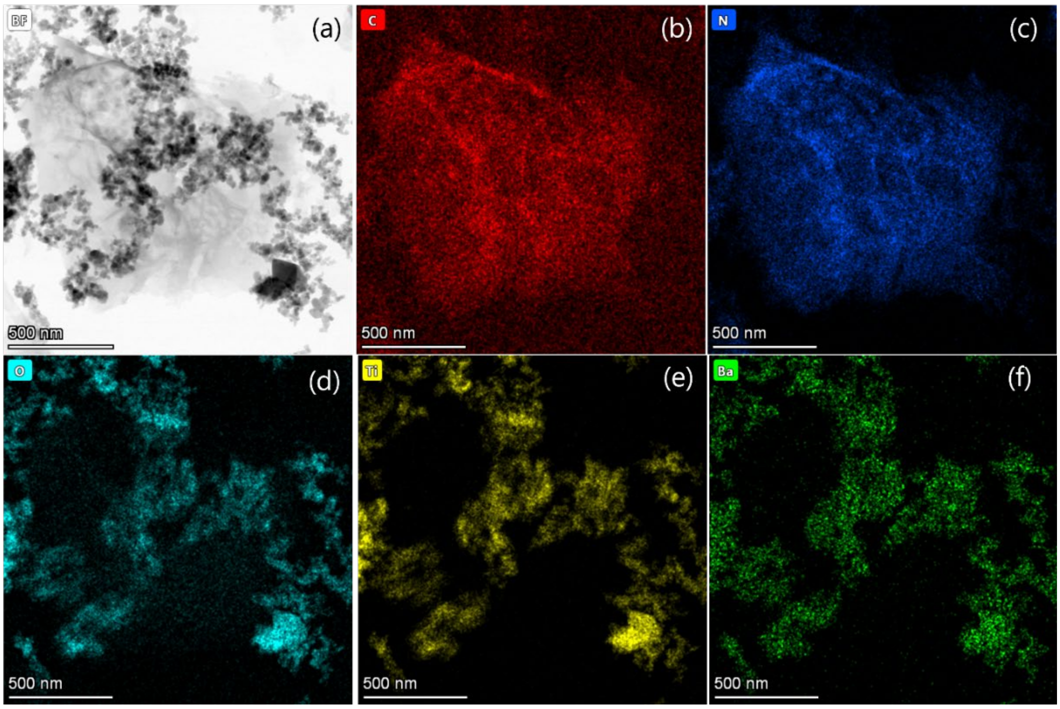

Fig. S3 TEM-EDS elemental mapping of CN-1BaTi. (a) TEM image, (b) carbon, (c) nitrogen, (d) oxygen, (e) titanium, and (f) barium.

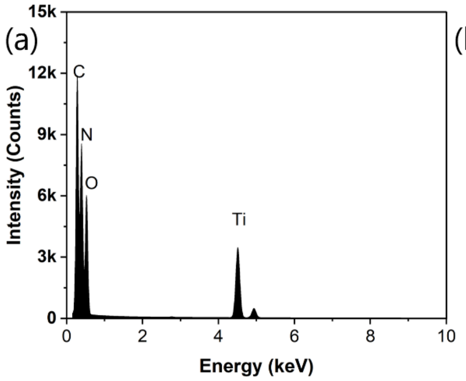

(b)

Element	Atomic fraction-%	Fit error
C	5.23298e+01	7.46678e-01
N	2.71506e+01	4.13385e+00
O	1.54282e+01	2.13850e+00
Ti	5.09140e+00	4.96724e-01
Ba	0.00000e+00	0.00000e+00

Fig. S4 (a) EDS spectra and (b) calculated atomic fraction of different elements of CN-1BaTi from Fig. S3.

Note: TEM-EDS can detect both Ba and Ti, but accurately distinguishing and quantifying their amounts is often challenging due to peak overlap. Specifically, Ba Lα (~4.47 keV) overlaps closely with Ti Kα (~4.51 keV), and Ba Lβ (~4.83 keV) is near Ti Kβ (~4.93 keV). As a result, the EDS detector may combine these signals, and the software can mis-attribute intensities, making accurate quantification unreliable without advanced peak deconvolution.

As shown in Fig. S3, the Ti and O elements are mainly concentrated in the nanoparticles. While the elemental mapping still detects the presence of Ba, the quantification results (Fig. S4)

suggest that the Ba content is negligible, and the nanoparticles are primarily composed of titanium oxide.

To accurately quantify the Ba content, complementary techniques are required. Therefore, we combined ICP and XRD analyses of CN-1BaTi, which both indicate a negligible amount of Ba and no detectable Ba-related phases. A small amount of Ba doping on the surface of the TiO_2 nanoparticles is possible, but we could not confirm this due to limited access to high-resolution TEM.

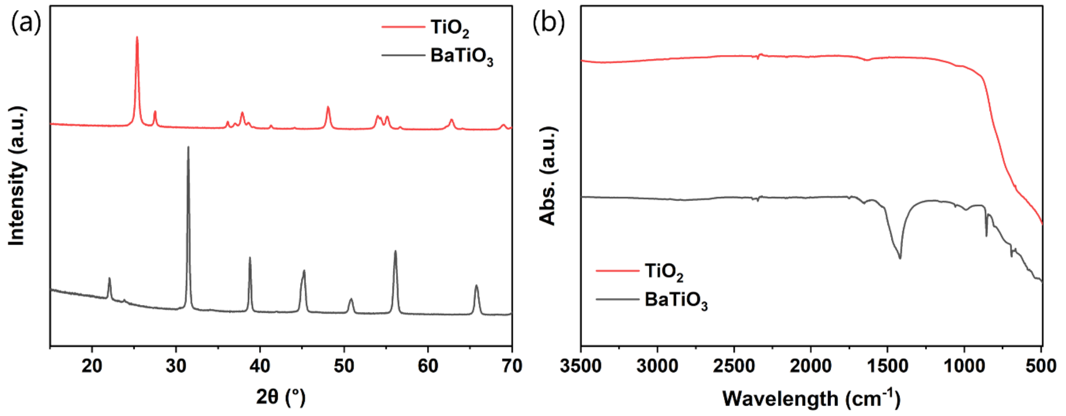

Fig. S5. (a) XRD patterns and (b) FTIR spectra for P25 (TiO_2) and BTO ($BaTiO_3$).

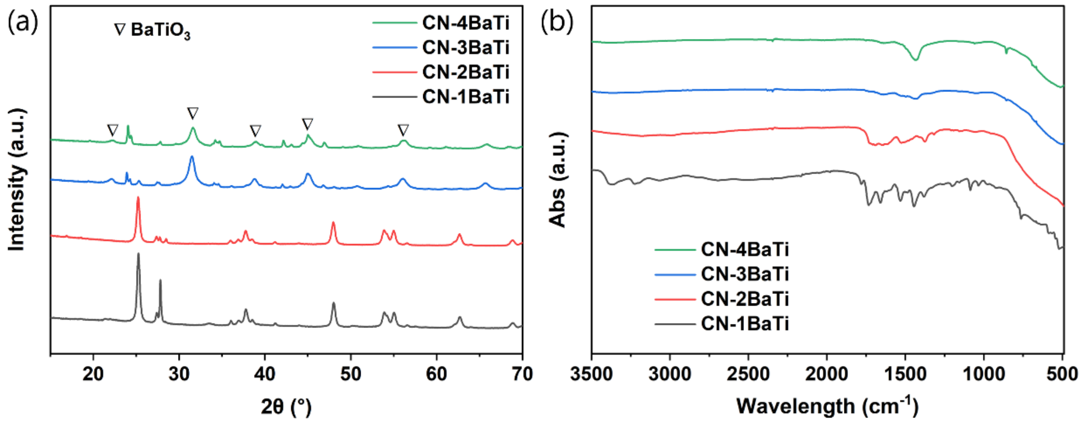

Fig. S6. (a) XRD patterns and (b) FTIR spectra for CN-2BaTi, CN-3BaTi, and CN-4BaTi.

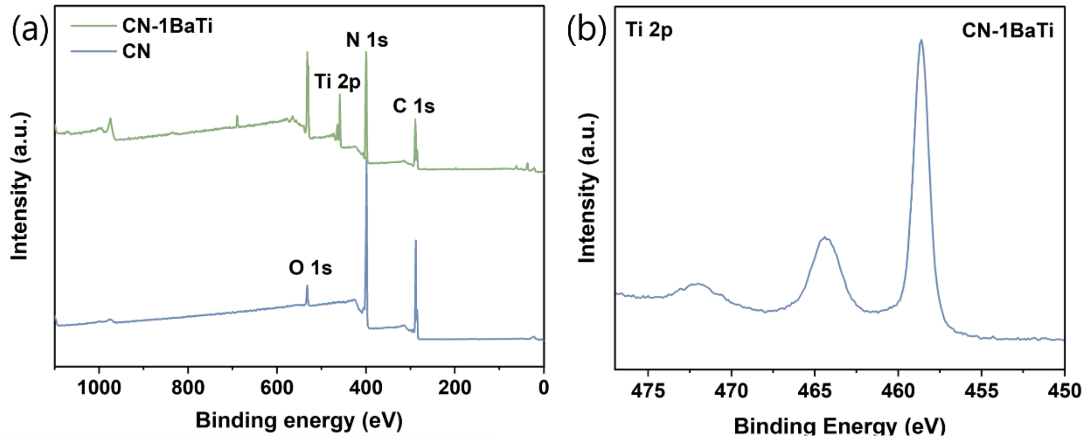

Fig. S7. (a) XPS survey scans for CN and CN-1BaTi, (b) Ti 2p high-resolution XPS of CN-1BaTi.

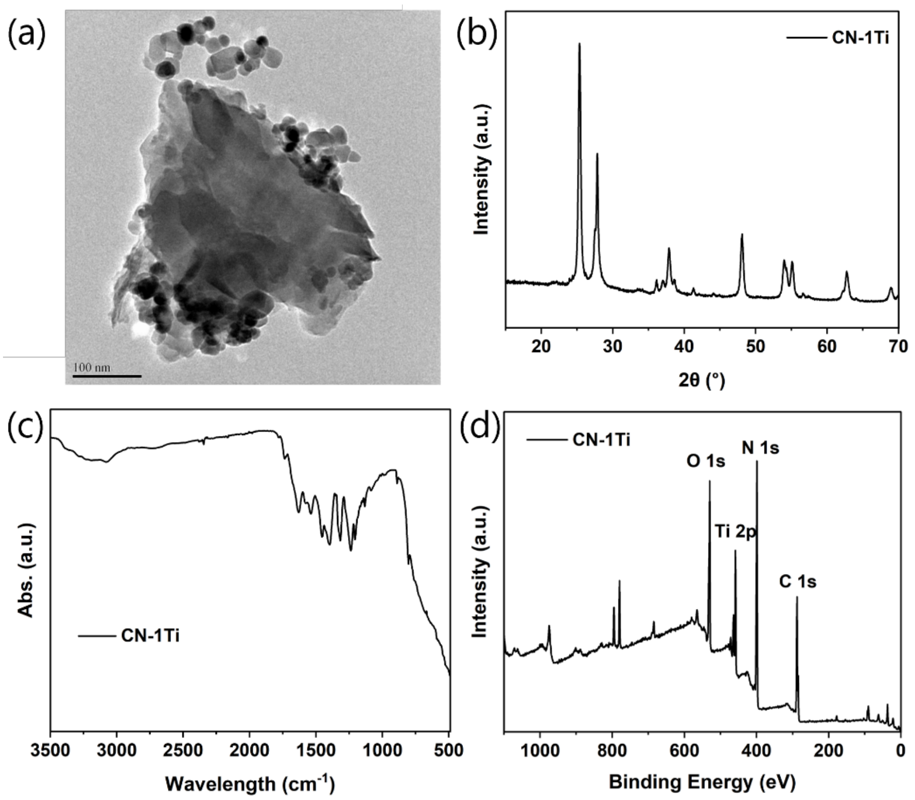

Fig. S8. (a) TEM image, (b) XRD pattern, (c) FTIR spectrum and (d) XPS survey scan for CN-1Ti.

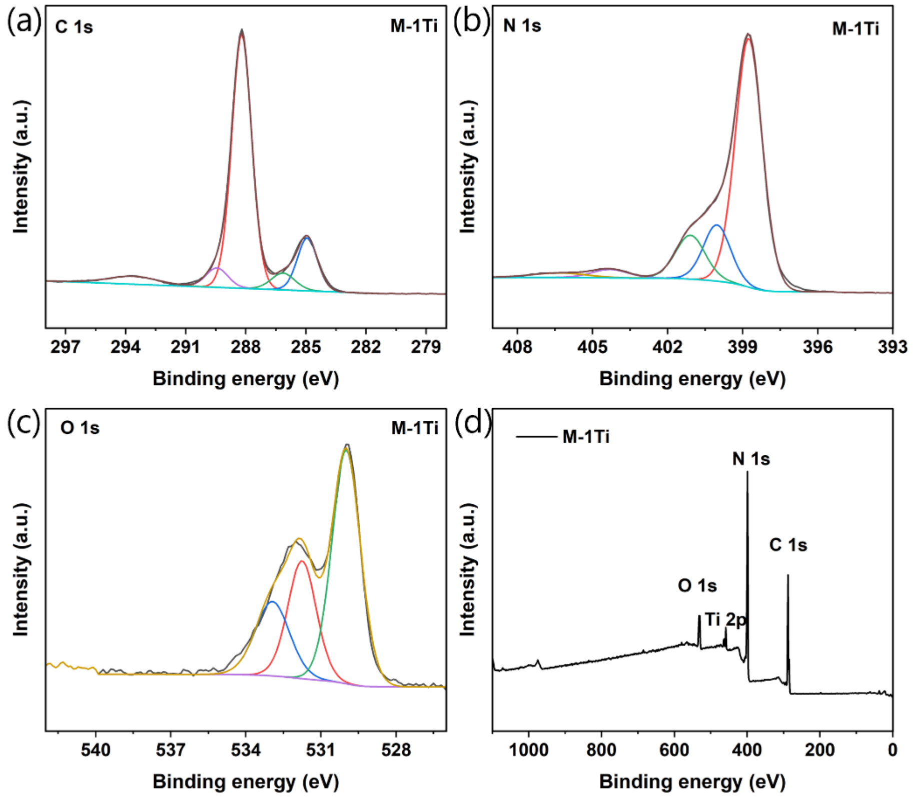

Fig. S9. (a) C 1s, (b) N 1s, (c) O 1s and (d) survey scan XPS spectra for M-1Ti.

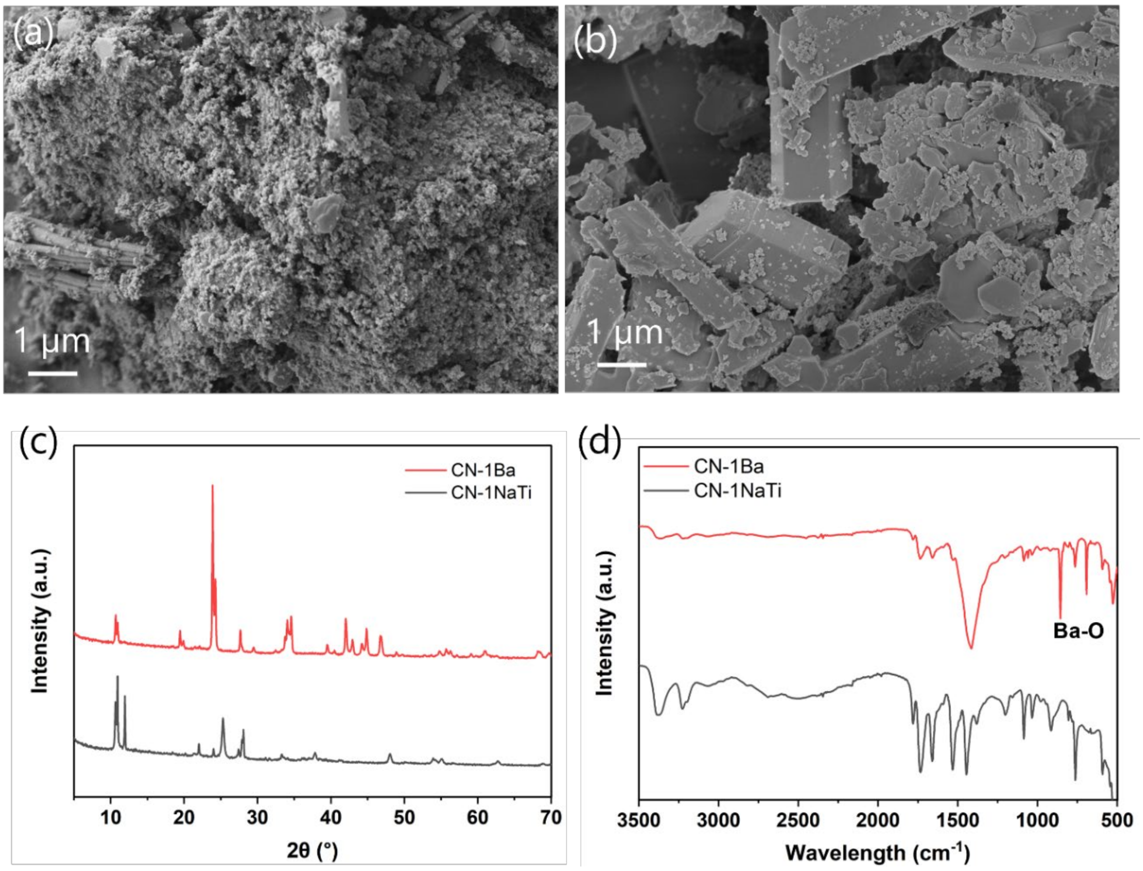

Fig. S10 SEM images of (a) CN-1NaTi and (b) CN-1Ba, (c) XRD patterns, and (d) FTIR spectra for CN-1Ba and CN-1NaTi.

The XRD diffraction pattern of CN-1NaTi indicates that the major phases are $Na_2Ti_3O_7$ (JCPDS no.48-0528) and $H_2Ti_3O_7$ (JCPDS no.36-0654), with a minor trace phase of Anatase TiO_2 (JCPDS no.21-1272). The pattern is complex with intermediate crystalline phases, and the overlapping of peaks resulted in peak shifts and peak broadening throughout the spectrum. However, these intermediates are a well-known consequence of multi-step $BaTiO_3$ syntheses reported in the literature [Chemosphere 338 (2023): 139337], and do not, by themselves, improve piezo-catalytic activity. The crystalline structure in CN-1Ba is mainly composed of the $Ba(OH)_2$ phase (JCPDS no. 00-045-1471). The $Ba(OH)_2$ does not exhibit piezocatalytic activity.

Rational design of the control samples:

• *g*-C_3N_4+NaOH (CN-1Na) and *g*-C_3N_4+NaOH+TiO_2 (CN-1NaTi) to isolate the effect of pH.

• *g*-C_3N_4+$Ba(OH)_2$ (CN-1Ba) to distinguish the distinct role of TiO_2 and the Ba^{2+}/OH^- in creating an oxidative hydrothermal environment.

• Pure $BaTiO_3$ (BTO) and composites with varying $BaTiO_3$ loading on *g*-C_3N_4 (CN-2BaTi, CN-3BaTi, and CN-4BaTi) to show that simply combining two piezoelectric materials does not yield a synergistic (>1+1) effect.

• A physical mixture of g-C_3N_4 and TiO_2 (M-1Ti) was synthesized under identical conditions to demonstrate the unique advantages of our one-pot hydrothermal approach.

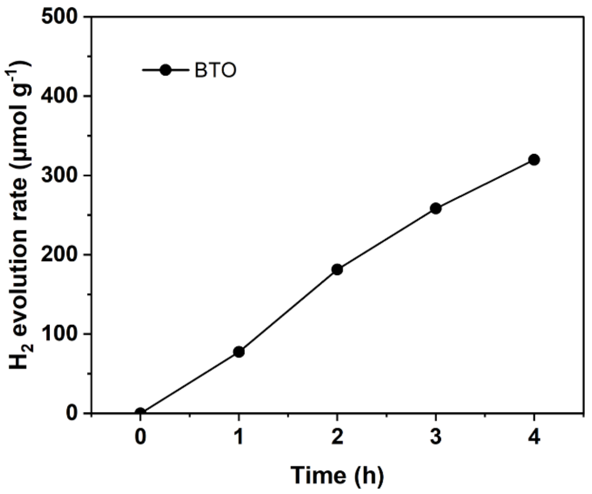

Fig. S11. H_2 evolution rate of BTO for 4 h under ultrasonication at 45 kHz and 80 W.

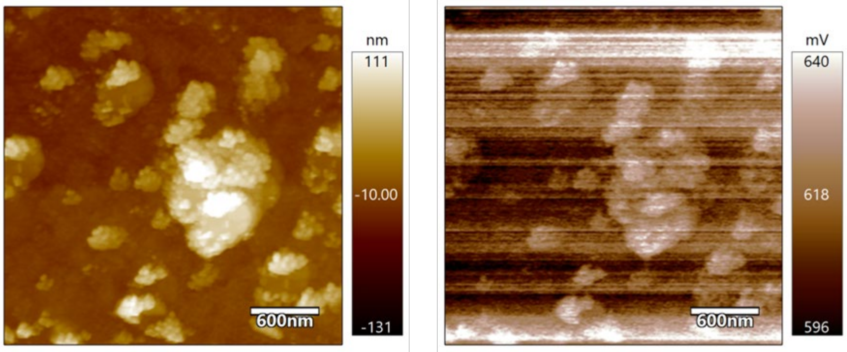

Fig. S12 The KPFM measurement results of CN-1BaTi flakes on ITO substrate: (a) Height, (b) surface potential distribution.

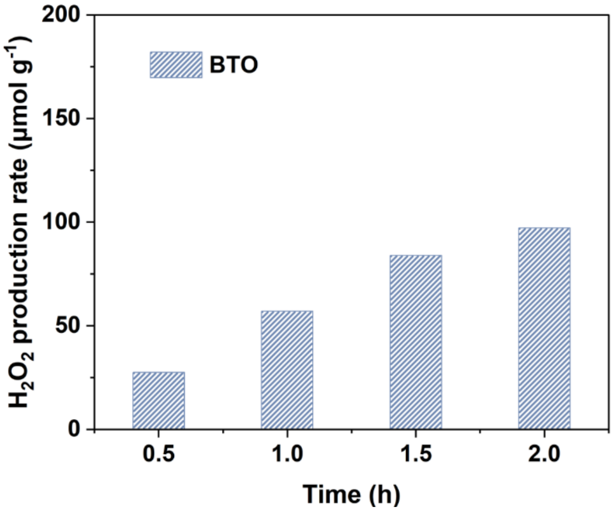

Fig. S13. H_2O_2 production rate of BTO for 2 h under ultrasonication at 45 kHz and 80 W.

Table S1. Carbon and nitrogen compositions are according to elemental analyzer in weight (wt.%), and metals are according to ICP in weight (wt.%).

Sample	Composition wt.%			ICP (wt.%)	
	C	N	H	Ba	Ti
CNNA	32.52	58.00	2.31	/	/
CNNA-1BTO	11.19	19.21	1.36	0.17	33.42
CNNA-2BTO	/	3.81	1.34	0.21	45.98
CNNA-3BTO	/	1.16	1.04	49.83	19.04
CNNA-4BTO	/	0.58	1.19	40.24	25.83

Table S2. Comparison of piezocatalytic activity among various carbon nitride-based materials.

Materials	External source	Production rate (μmol g^{-1} h^{-1})		Additives	Ref.
		H_2	H_2O_2		
Cv/Nv-C_3N_4	53 kHz, 150 W	/	680	/	[1]
UT-*g*-C_3N_4	40 kHz, 240 W	8350	3880	Glucose	[2]
N-TiO_2@C NSs	45 kHz, 200 W	390	/	/	[3]
g-C_3N_4/*g*-C_3N_4	40 kHz, 100 W	/	35.4	/	[4]
P-C_3N_4/PVDF-HFP	40 kHz, 300 W	/	33.43	Ethanol	[5]
C_5N_2	40 kHz, 400 W	/	480.1	/	[6]
**CN-1BT**	**45 kHz, 80 W**	**4427.2**	**809.3**	**/**	**Ours**

References

[1] K. Wang, D. Shao, L. Zhang, Y. Zhou, H. Wang, W. Wang, Efficient piezo-catalytic hydrogen peroxide production from water and oxygen over graphitic carbon nitride, J. Mater. Chem. A 7 (2019) 20383-20389. https://doi.org/10.1039/c9ta06251c.

[2] C. Hu, F. Chen, Y. Wang, N. Tian, T. Ma, Y. Zhang, H. Huang, Exceptional Cocatalyst-Free Photo-Enhanced Piezocatalytic Hydrogen Evolution of Carbon Nitride Nanosheets from Strong In-Plane Polarization, Adv Mater 33 (2021). https://doi.org/10.1002/adma.202101751.

[3] X. Zhao, X. Lu, W.J. Chen, M.Q. Yang, X. Pan, Z. Bian, Exceptional piezocatalytic H2 production of nitrogen-doped TiO_2@carbon nanosheets induced by engineered piezoelectricity, J Colloid Interface Sci 659 (2024) 11-20. https://doi.org/10.1016/j.jcis.2023.12.101.

[4] H. Zhai, H. Liu, Y. Zhang, J. Tong, X. Liu, W. Du, H. Liao, P. Tan, J. Pan, Flexible construction of heteroatom-free *g*-C_3N_4/*g*-C_3N_4 homojunction with switching charge dynamics

toward efficient photo-piezocatalytic performance, Appl Catal B 349 (2024) 123909. https://doi.org/10.1016/j.apcatb.2024.123909.

[5] Z. Chen, J. Zhuang, C. Liu, M. Chai, S. Zhang, K. Teng, T. Cao, Y. Zhang, Y. Hu, L. Zhao, Q. An, Effective H_2O_2 Production via Favorable Intermediate Desorption in Fluctuating Electrical Fields from Matrix-Filler Mutually Enhanced P-C_3N_4/PVDF-HFP Porous Composite, ChemElectroChem 9 (2022). https://doi.org/10.1002/celc.202200124.

[6] J. Ma, C. Peng, X. Peng, S. Liang, Z. Zhou, K. Wu, R. Chen, S. Liu, Y. Shen, H. Ma, Y. Zhang, H_2O_2 Photosynthesis from H_2O and O_2 under Weak Light by Carbon Nitrides with the Piezoelectric Effect, J Am Chem Soc 146 (2024) 21147–21159. https://doi.org/10.1021/jacs.4c07170.
